# FOXC2 Alleviates Myocardial Ischemia-Reperfusion Injury in Rats through Regulating Nrf2/HO-1 Signaling Pathway

**DOI:** 10.1155/2021/9628521

**Published:** 2021-11-23

**Authors:** Rui Wang, Yonggang Wu, Shoutao Jiang

**Affiliations:** Department of Cardiology, Guangzhou Hospital of Integrated Chinese and Western Medicine, Guangzhou, China

## Abstract

**Objective:**

Myocardial ischemia-reperfusion injury (MIRI) is the leading cause of death in patients with cardiovascular disease. The purpose of this study is to investigate the effect and mechanism of forkhead box C2 (FOXC2) on MIRI in rats.

**Methods:**

We made ischemia-reperfusion (I/R) models for rats by performing I/R surgery. After 3 hours, 3 days, and 7 days of reperfusion, we detected the structure and function of rat myocardium by 2, 3, 5-triphenyl tetrazolium chloride staining, echocardiography, lactate dehydrogenase kit, and haematoxylin-eosin staining. The change of FOXC2 expression in myocardial tissue was also detected. Then, we increased the expression of FOXC2 in rats by adenovirus transfection to clarify the effect of FOXC2 on changes of oxidative stress and inflammation of rat myocardium. In addition, we detected the effect of FOXC2 overexpression plasmid on the function of H9c2 cells *in vitro*. The expression changes of Nrf2/HO-1 in myocardial cells were also detected to clarify the mechanism of action of FOXC2.

**Results:**

The expression of FOXC2 in I/R rats was significantly lower than that in the sham group. After overexpressing FOXC2 in I/R rats, we found that the expression of SOD1/2 of rat myocardium and inflammatory factors in the serum were significantly reduced. Overexpression of FOXC2 also increased the viability and antioxidant capacity of H9c2 cells. In addition, FOXC2 was found to increase the activity of the Nrf2/HO-1 signaling pathway in myocardial cells, and the inhibition of Nrf2/HO-1 signaling pathway attenuated the protective effect of FOXC2 on myocardial cells.

**Conclusions:**

MIRI in rats was accompanied by low expression of FOXC2 in myocardial tissue. Overexpression of FOXC2 reduces the level of inflammation and oxidative stress in myocardial tissue by promoting the Nrf2/HO-1 signaling pathway, thereby alleviating MIRI.

## 1. Introduction

Acute myocardial infarction is the acute manifestation of coronary heart disease and the main cause of death. Its basic pathological changes include rupture of coronary plaques, thrombosis, and decreased blood supply, which eventually leads to severe ischemia in myocardial tissues [1]. Timely reperfusion therapy, such as drug thrombolysis, percutaneous coronary intervention, and coronary artery bypass grafting treatment, is beneficial to recanalize occlusive vessels as early as possible and reduce infarct size [2]. However, with further research on the process of myocardial ischemia and reperfusion, the researchers found that although reperfusion therapy can make the ischemic heart regain blood perfusion in a short period of time, the reperfusion therapy itself can also lead to severe dysfunction and structural injury [3]. This phenomenon is called myocardial ischemia-reperfusion injury (MIRI). After myocardial ischemia reperfusion, although the blood supply is restored, the increase in the generation of oxygen free radicals of myocardial cells and the overload of calcium ions during ischemia will initiate the programmed apoptosis of myocardial cells, resulting in structural and functional damage of myocardial cells [4].

Forkhead box C2 (FOXC2) belongs to the forkhead transcription factor family [5]. The role of FOXC2 was first confirmed in lymphedema-distichiasis syndrome. FOXC2 protein is expressed in various tissues such as bone, fat, and tumor and plays an important role in the development of the cardiovascular system, lymphatic system, and axial bone system [6]. A study showed that FOXC2 can regulate angiopoietin-like protein 2, thereby affecting the level of apoptosis and inflammation of macrophages [7]. FOXC2 has also been found to promote bone marrow mesenchymal differentiation by regulating Wnt-*β*-catenin signaling pathway [8]. In addition, Kume et al. revealed that FOXC1 and FOXC2 are both required for cardiovascular development and somitogenesis [9]. However, the role of FOXC2 in MIRI is still unclear. Therefore, we used rats to make MIRI models to detect changes in FOXC2 expression in myocardial tissue. Then, we studied the effect of FOXC2 on the MIRI of rats by adenovirus transfection.

## 2. Materials and Methods

### 2.1. Animals and Grouping

Sixty male Sprague Dawley rats (8-10 weeks, 180-200 g) were used in this study. All rats were provided by Guangzhou Hospital of Integrated Chinese and Western Medicine Animal Center and housed in specific pathogen-free animal rooms. The room temperature of the animal rooms was 22-24°C, and the relative humidity was 50-60%. We used rats to make the ischemia-reperfusion (I/R) model and detected the structure and function of the rat heart after 3 hours, 3 days, and 7 days. This study was approved by the Animal Ethics Committee of Guangzhou Hospital of Integrated Chinese and Western Medicine Animal Center.

### 2.2. Procedure of Ischemia-Reperfusion (I/R) Model

After anesthetizing the rat with 2% sodium pentobarbital (40 mg/kg), we fixed the rat on the operating table and removed the fur from rat's chest. We cut the skin and trachea of rat's neck and then use the animal ventilator (CWE SAR-830, Orange, CA, USA) to maintain rat's breathing. The ventilator was set to the heart rate of 80-100 times/min, the respiratory rate of 60 times/min, the breathing ratio of 1 : 1.5, and the tidal volume of 70 mL. Then, we cut the skin of rat's left chest and bluntly separated the muscles. After the heart was exposed, we cut the pericardium and ligated the left anterior descending coronary artery. The cyanosis of the epicardium distal to the ligation site and the ST segment elevation of the electrocardiogram indicated myocardial ischemia. After 30 minutes, we loosened the ligature. Hyperemia of myocardial tissue and the ST segment depression of the electrocardiogram indicated successful myocardial reperfusion [10]. We measured cardiac structure and function at 3 hours, 3 days, and 7 days after reperfusion.

### 2.3. Adenovirus Transfection

Seven days before making the I/R model, we fixed the rats on the operating table. Then, we wiped the tail vein of the rats with 75% alcohol to fully dilate the tail vein. We used a microsyringe (Molecular Devices, Santa Clara Valley, MD, USA) to inject 20 *μ*L of purified adenovirus (Genepharma, Shanghai, China), including negative control (NC) adenovirus and FOXC2 overexpressing adenovirus, into the tail vein of rats. Adenovirus was constructed in Genepharma (Genepharma, Shanghai, China) with the titer of 1 × 10^11^ plaque forming unit.

### 2.4. 2, 3, 5-Triphenyl Tetrazolium Chloride (TTC) Staining

We collected rat hearts and rinsed them with normal saline. Then, we put the rat hearts in the -20°C refrigerator for 20 minutes. The heart tissue was cut into 5 pieces with the thickness of 2 mm. Then, we put the slices in 1% TTC staining solution (Sigma-Aldrich, St. Louis, MO, USA) for 15 minutes. Finally, we used the camera to take pictures and analyze the results. Normal tissues appeared red, while ischemic tissues appeared pale. Area at risk (AAR) was expressed as the percentage of the left ventricle.

### 2.5. Echocardiography

We tilted rat's body 30° to the left and placed the ultrasound probe on rat's left chest. Left ventricular ejection fraction (LVEF), left ventricular fractional shortening (LVFS), left ventricular end-diastolic volume (LVEDV), left ventricular end-systolic volume (LVESV), left ventricular end-diastolic diameter (LVEDd), and left ventricular end-systolic diameter (LVESd) were detected.

### 2.6. Detection of Lactate Dehydrogenase (LDH)

We took 2 mL of rat aortic blood and separated the serum. The LDH kit (R&D Systems, Emeryville, CA, USA) was used to detect the LDH level in serum. Matrix buffer and 5 *μ*L of coenzyme 1 were added to the serum. We placed the mixture in a 37°C incubator for 15 minutes. Then, we added 25 *μ*L of 2, 4-dinitrophenylhydrazine and continued to incubate for 15 minutes. Finally, we added 250 *μ*L of 0.4 M NaOH and measured the absorbance of the mixture at 450 nm with a spectrophotometer. The same method was also used to detect LDH level in H9c2 cells.

### 2.7. Enzyme Linked Immunosorbent Assay (ELISA)

We used ELISA kits (R&D Systems, Emeryville, CA, USA) to detect the levels of inflammatory factors, interleukin (IL)-1*β*, and tumor necrosis factor (TNF)-*α* in rat serum. Standards were used to make standard curves. Then, we detected the absorbance of the sample and calculated the concentration of the sample according to the standard curve.

### 2.8. Haematoxylin-Eosin (HE) Staining

We collected rat hearts and fixed them with 4% paraformaldehyde. Then, we made them into paraffin blocks and used a microtome to cut the paraffin block into paraffin slices. HE staining was used to detect the morphology of rat myocardial tissue. We used xylene for dewaxing and alcohol solution for hydration. Then, we stained the cell nucleus with hematoxylin (Beyotime, Shanghai, China) and stained the cytoplasm with eosin (Beyotime, Shanghai, China). Finally, we used an optical microscope to observe the morphology of myocardial tissue.

### 2.9. Immunohistochemical (IHC) Staining

We used xylene and alcohol solutions for dewaxing and hydration. Then, we used citrate buffer to repair the antigen. 3% H_2_O_2_ was used to remove peroxidase. We then used 10% goat serum to block nonspecific antigens. After adding the primary antibody dilution (FOXC2, ab65141; SOD1, ab13498; SOD2, ab13533. Abcam, Cambridge, MA, USA) to myocardial tissue, we placed the slices in a 4°C refrigerator overnight. Then, we used secondary antibody dilution (Abcam, Cambridge, MA, USA) to incubate myocardial tissue and used diaminobenzidine (DAB) for color development. Finally, we used an optical microscope to observe the staining results.

### 2.10. Cell Culture

The rat myocardial cell line, H9c2 cells, was used in this study. Dulbecco's modified eagle medium (Gibco, Rockville, MD, USA) containing 10% fetal bovine serum (Gibco, Rockville, MD, USA) and 1% penicillin plus streptomycin (Gibco, Rockville, MD, USA) was used to culture H9c2 cells. H9c2 cells were cultured in an incubator with 37°C and 5% CO_2_.

### 2.11. Procedure of Hypoxia Reoxygenation (H/R) Model

The H/R model was used to simulate rat I/R *in vitro*. After washing the cells with PBS, we added simulated ischemic fluid and placed the cells in an incubator with 95% N_2_ and 5% CO_2_ for 10 hours. Then, we recultivated the cells under normal conditions for 24 hours.

### 2.12. RNA Isolation and Quantitative Real-Time Reverse Transcription-Polymerase Chain Reaction (RT-PCR)

TRIzol reagent (Invitrogen, Carlsbad, CA, USA) was used to extract total RNA from rat myocardium and H9c2 cells. We used a spectrophotometer to detect the concentration of total RNA. We used the reverse transcription kit (Vazyme, Nanjing, China) to configure the reverse transcription system according to manufacturer's instructions and reversed the mRNA to complementary deoxyribose nucleic acid (cDNA) using a PCR machine. Then, we used SYBE Green Master Mix (Vazyme, Nanjing, China) and primers to amplify the cDNA. Primer sequences were shown in Table 1. The expression level of glyceraldheyde 3-phosphate dehydrogenase (GAPDH) was used as reference. The ratio of the RNA concentration of each group to the sham group or control group was used to represent the relative concentration of RNA (2^−ΔΔCt^).

### 2.13. Plasmid Transfection

We seeded H9c2 cells into six-well plates. When the cell growth density reached 30%, we used Lipofectamine 3000 reagent (Sigma-Aldrich, St. Louis, MO, USA) to transfect NC plasmid and FOXC2 overexpression plasmid into H9c2 cells. RT-PCR was used to detect the transfection efficiency.

### 2.14. Cell Counting Kit-8 (CCK-8) Assay

The CCK-8 assay was used to detect the viability of H9c2 cells. We used 96-well plates to culture H9c2 cells with 5000 cells per well. After treating the cells, we added 10 *μ*L of CCK-8 reagent (Dojindo Molecular Technologies, Kumamoto, Japan) to each well. After putting the cells into the incubator for 2 hours, we used a microplate reader to detect the absorbance of each well at 450 nm.

### 2.15. Immunofluorescent (IF) Staining

We used 24-well plates to culture cells. Then, we discarded the medium and fixed the cells with 4% paraformaldehyde. Then, we used 0.2% TritonX-100 to treat the cells for 15 minutes. 10% goat serum was used to block nonspecific antigens. We used primary antibody dilution (SOD1, ab13498; SOD2, ab13533; Nrf2, ab31163; HO-1, ab13248. Abcam, Cambridge, MA, USA) to incubate cells at 4°C overnight and fluorescent secondary antibody dilution (Abcam, Cambridge, MA, USA) to incubate cells at room temperature. After staining the cell nucleus with 4′,6-diamidino-2-phenylindole (DAPI), we used a fluorescence microscope to observe the staining results.

### 2.16. Statistical Analysis

Statistical Product and Service Solutions 20.0 statistical software (IBM, Armonk, NY, USA) was used to analyze the results of this study. All data were represented as mean ± standard deviation. Differences between two groups were analyzed by using Student's *t*-test. Comparison between multiple groups was done using one-way ANOVA test followed by post hoc test (least significant difference). *P* < 0.05 indicated that the difference was statistically significant. All experiments were repeated 3 times.

## 3. Results

### 3.1. Expression of FOXC2 Decreased in Myocardial Tissue after I/R

**Figure 1:**
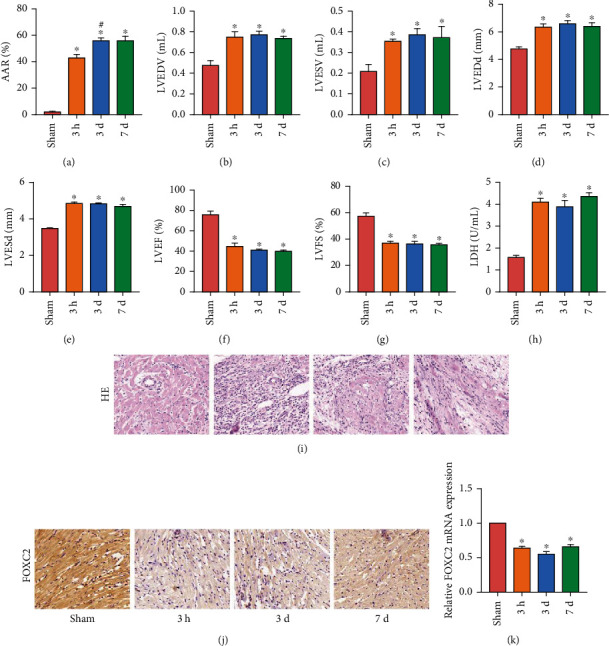
Expression of FOXC2 decreased in myocardial tissue after I/R. (a) AAR of rats; (B–G) cardiac function (LVEDV, LVESV, LVEDd, LVESd, LVEF, and LVFS) in rats; (h) LDH level of rat serum; (i) HE staining of rat myocardium (200x); (j) IHC staining results of FOXC2 expression of rat myocardium (200x); (k) mRNA expression of FOXC2 in rat myocardium (“^∗^” means *P* < 0.05 vs. sham group; “^#^” means *P* < 0.05 vs. 3 d group).

After making the I/R model for rats, we reperfused them for 3 hours, 3 days, and 7 days and collected the rat heart tissue for TTC staining to observe the changes of rat AAR (Figure 1(a)). After reperfusion, the AAR of rat myocardium increased significantly, and the AAR after 3 days was greater than that after 3 hours. There was no significant difference between AAR after 7 days and AAR after 3 days. In addition, we detected changes in rat cardiac function by echocardiography. After I/R, LVEDV (Figure 1(b)), LVESV (Figure 1(c)), LVEDd (Figure 1(d)), and LVESd (Figure 1(e)) of rats increased significantly, while LVEF (Figure 1(f)) and LVFS (Figure 1(g)) decreased. We also detected the concentration of myocardial injury marker LDH in the serum of rats and found that the concentration of LDH was increased in the serum of the I/R rats (Figure 1(h)). The results of HE staining also showed that the structure of myocardial tissue of I/R rats was disordered (Figure 1(i)). These results indicated that the I/R model was successfully established. We detected the FOXC2 in rat myocardial tissue by IHC staining (Figure 1(j)) and RT-PCR (Figure 1(k)). After I/R, the expression of FOXC2 mRNA and protein in rat myocardium was significantly reduced.

### 3.2. Overexpression of FOXC2 Reduced Inflammation and Oxidative Stress in I/R Rat Myocardium

**Figure 2:**
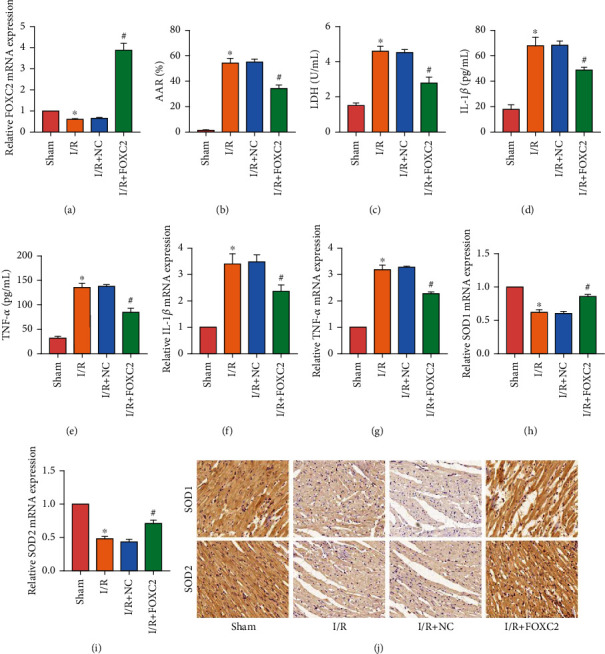
Overexpression of FOXC2 reduced inflammation and oxidative stress in I/R rat myocardium. (a) mRNA expression of FOXC2 in rat myocardium; (b) AAR of rats; (c) LDH level of rat serum; (d, e) ELISA results of IL-1*β* and TNF-*α* in rat serum; (f–i) mRNA expression of IL-1*β*, TNF-*α* SOD1, and SOD2 in rat myocardium. (j) IHC staining results of SOD1/2 expression of rat myocardium (200x) (“^∗^” means *P* < 0.05 vs. sham group; “^#^” means *P* < 0.05 vs. I/R+NC group).

To detect the effect of FOXC2 on MIRI, we used adenovirus to overexpress FOXC2 in rats. The rats with reperfusion for 3 hours showed significant myocardial injury, so we took the rats with reperfusion for 3 hours as the study object. The rats were divided into sham group, I/R group, I/R + negative control (NC) group, and I/R + FOXC2 group. Rats in the I/R + NC group and the I/R + FOXC2 group were injected with NC and FOXC2 overexpressing adenovirus from the tail vein one week before the I/R model was made. RT-PCR detected the expression of FOXC2 mRNA in rat myocardial tissue and found that FOXC2 adenovirus effectively increased the expression of FOXC2 in rat myocardial tissue (Figure 2(a)). We detected the degree of myocardial injury in rats by TTC staining (Figure 2(b)) and LDH (Figure 2(c)) measurement. After overexpressing FOXC2, AAR and LDH of rats were significantly reduced. We detected the expression of inflammatory factors IL-1*β* and TNF-*α* in rat serum by ELISA (Figures 2(d) and 2(e)) and RT-PCR (Figures 2(f) and 2(g)). FOXC2 has been found to reduce inflammation level in rats. In addition, we determined the change of oxidative stress level in rat myocardial tissue by detecting the expression of superoxide dismutase (SOD)1/2 in myocardium. The results of RT-PCR (Figures 2(h) and 2(i)) and IHC staining (Figure 2(j)) showed that overexpression of FOXC2 effectively increased the expression of SOD1/2 in rat myocardium.

### 3.3. Overexpression of FOXC2 Alleviated H/R-Induced H9c2 Cell Injury by Promoting the Nuclear Factor-Erythroid 2-Related Factor 2 (Nrf2)/Heme Oxygenase (HO)-1 Signaling Pathway

**Figure 3:**
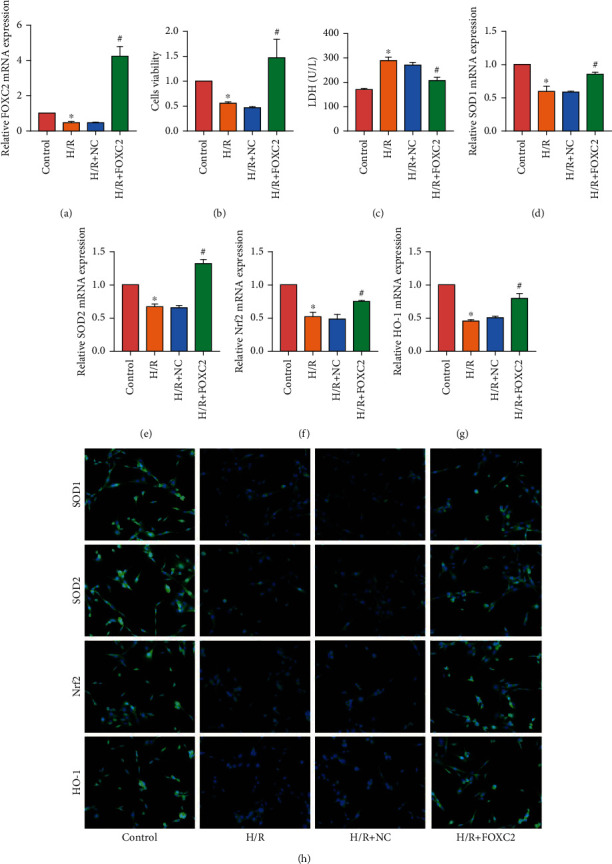
Overexpression of FOXC2 alleviated H/R-induced H9c2 cell injury by promoting the Nrf2/HO-1 signaling pathway. (a) mRNA expression of FOXC2 in H9c2 cells; (b) CCK-8 assay results of H9c2 cells; (c) LDH level of H9c2 cells; (d–g) mRNA expression of SOD1/2, Nrf2, and HO-1 in H9c2 cells; (h) IF staining results of SOD1/2, Nrf2, and HO-1 in H9c2 cells (200x) (“^∗^” means *P* < 0.05 vs. control group; “^#^” means *P* < 0.05 vs. H/R+NC group).

We induced H9c2 cell injury through H/R and transfected cells with FOXC2 overexpression plasmid. RT-PCR results showed that FOXC2 overexpression plasmid significantly increased FOXC2 expression in H9c2 cells (Figure 3(a)). The CCK-8 assay detected the viability of H9c2 cells. The viability of H9c2 cells decreased significantly after H/R, and the overexpression of FOXC2 could increase the viability of H9c2 cells (Figure 3(b)). We examined the level of LDH in H9c2 cells and found that overexpression of FOXC2 can reduce the LDH level (Figure 3C). In addition, we examined the effect of FOXC2 on the level of oxidative stress in H9c2 cells. RT-PCR and IF staining found that FOXC2 can promote the expression of SOD1/2, Nrf2, and HO-1 in H9c2 cells (Figures 3(d)–3(h)).

### 3.4. Inhibition of Nrf2/HO-1 Signaling Pathway Attenuated the Protective Effect of FOXC2 on H9c2 Cells

**Figure 4:**
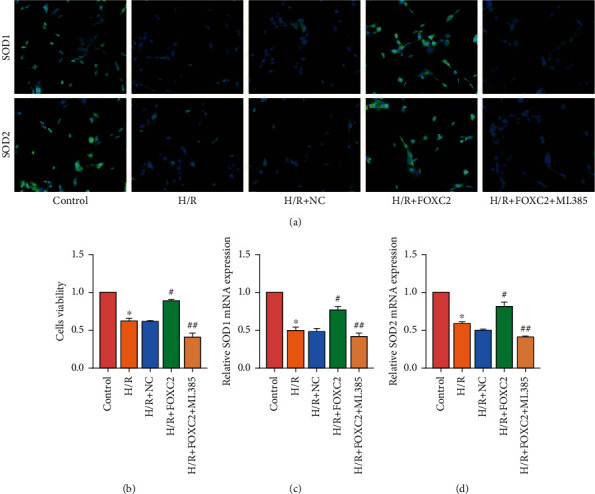
Inhibition of Nrf2/HO-1 signaling pathway attenuated the protective effect of FOXC2 on H9c2 cells. (a) IF staining results of SOD1/2 in H9c2 cells (200x); (b) CCK-8 assay results of H9c2 cells; (c, d) mRNA expression of SOD1/2 in H9c2 cells (“^∗^” means *P* < 0.05 vs. control group; “^#^” means *P* < 0.05 vs. H/R+NC group; “^##^” means *P* < 0.05 vs. H/R+FOXC2 group).

In order to verify the mechanism of FOXC2 protecting myocardial cells, we used Nrf2/HO-1 signaling pathway inhibitor ML385 to treat H9c2 cells based on FOXC2 transfection. The results of IF staining showed that after treatment of H9c2 cells with ML385, the expression of SOD1/2 in H9c2 cells was reduced, indicating that the antioxidant capacity of the cells was reduced (Figure 4(a)). The CCK-8 assay also found that ML385 attenuated the promoting effect of FOXC2 on cell viability (Figure 4(b)). RT-PCR results also showed that ML385 reduced the expression of SOD1/2 mRNA in H9c2 cells (Figures 4(c) and 4(d)).

## 4. Discussion

MIRI is the pathophysiological disorder that mainly occurs during the transition from the ischemic phase to the reperfusion phase [11]. The mechanism of MIRI has not been fully elucidated, but a large number of studies have shown that MIRI may be involved in excessively generated oxygen free radicals, cytoplasmic and mitochondrial calcium overload, endothelial cell dysfunction, inflammation, and myocardial necrosis and apoptosis [12–14]. FOXC2, as a multifunctional cytokine *in vivo*, was found to play an important role in MIRI [15]. We found that the expression of FOXC2 in the rat myocardium group after I/R was reduced. After using adenovirus to increase the expression of FOXC2 in rat myocardium, the activity of SOD1/2 in rat myocardium increased, and the expression of inflammatory factors decreased, indicating that FOXC2 can reduce the level of oxidative stress and inflammation in rats. After increasing the expression of FOXC2 in H9c2 cells by plasmid transfection, we also found that FOXC2 can relieve H/R-induced myocardial cells injury *in vitro*. FOXC2 has been found to activate the Nrf2/HO-1 signaling pathway, which may allow FOXC2 to protect myocardial cells.

Studies have shown that the inflammatory response is activated during the myocardial ischemia stage, and reperfusion further exacerbates the inflammatory response of the myocardium [16]. Inflammation mainly caused by neutrophil infiltration is one of the important mechanisms. In addition, a clinical study found that after revascularization of myocardial infarction, inflammatory factors in patients' serum including TNF-*α*, IL-1*β*, IL-6, and IL-8 were significantly increased [17]. Niermann et al. [18] used oligophrenin1 to treat I/R rats and found that oligophrenin1 reduced myocardial cells apoptosis by regulating the inflammation level of myocardial tissue, thereby alleviating MIRI. Another study found that recombinant human IL-1*β* receptor inhibitors can alleviate myocardial injury in I/R rats by inhibiting IL-1*β* activity [19]. After treating I/R rats with FOXC2 overexpression adenovirus, we found that the expression of inflammatory factors in rat serum and myocardial tissue was significantly reduced through ELISA and RT-PCR.

Previous studies have shown that the imbalance between oxidation and antioxidation caused by myocardial ischemia can aggravate the damage of cardiac function and the degree of inflammatory response [20]. The increase of oxygen free radicals and the decrease of the activity of free radical scavenging enzymes are the direct causes of oxidative stress damage [21]. SOD, as a key enzyme for scavenging oxygen free radicals in the body, can directly reflect the level of oxygen free radicals in the body [22]. Sedova et al. [23] examined the level of oxidative stress in I/R rats and found that SOD activity in I/R rats was significantly lower than that in normal rats. After the treatment of rats with the antioxidant melatonin, the cardiac function of the rats was significantly improved. Loor et al. [24] found that oxidative damage to mitochondria is an important factor in cell death of MIRI. Mitochondria in ischemic myocardial cells produce reactive oxygen species through Bax/Bak-independent pathways, triggering mPTP activation, mitochondrial depolarization, and cell death. The FOXC family has been found to alleviate oxidative damage in rat models of chronic obstructive pulmonary disease [25]. FOXC2 has also been found to be related to oxidative stress-related pathways in cancer research [26]. We detected the expression of SOD1/2 in rat myocardial tissue and H9c2 cells by IHC staining and RT-PCR and found that FOXC2 can promote the activity of SOD1/2, thereby improving the antioxidant capacity of cardiomyocytes. Nrf2/HO-1 is an important pathway for the body to resist oxidative stress. When oxidative stress occurs in the body, the Nrf2 gene is activated and transcribed, which initiates the expression of downstream antioxidant proteins, oxidases, and phase II detoxification enzymes [27]. We found that FOXC2 can promote the expression of Nrf2 and HO-1 in H9c2 cells, and Nrf2/HO-1 signaling pathway inhibitor ML385 attenuated the protective effect of FOXC2 on H9c2 cells, suggesting that the activation of Nrf2/HO-1 may be an important mechanism by which FOXC2 protected myocardial cells. To our knowledge, this is the first study to investigate the effect of FOXC2 on MIRI. We hope to provide new targets for clinical MIRI treatment through this study.

## 5. Conclusions

After I/R, the expression of FOXC2 in the myocardium of rats was significantly reduced. Overexpression of FOXC2 reduced the level of inflammation and oxidative stress in myocardial tissue. In addition, FOXC2 promoted the activity of Nrf2/HO-1 signaling pathway in myocardial cells, and the inhibition of Nrf2/HO-1 signaling pathway attenuated the protective effect of FOXC2 on myocardial cells, indicating that FOXC2 alleviated MIRI by regulating the Nrf2/HO-1 pathway.

## Figures and Tables

**Table 1:** RT-PCR primers sequences.

Name	Sense/antisense (S/AS)	Sequence(5′-3′)
FOXC2	S	CACAGCGGGGACCTGAA
	AS	CAGCCGGTGGGAGTTGA

SOD1	S	CAATGTGGCTGCTGGAA
	AS	TGATGGAATGCTCTCCTGA

SOD2	S	GCCGTGTTCTGAGGAGAG
	AS	GTCGTAAGGCAGGTCAGG

Nrf2	S	ATTCCCAGCCACGTTGAGAG
	AS	TCCTGCCAAACTTGCTCCAT

HO-1	S	CCATCCCTTACACACCAGCC
	AS	GCGAGCACGATAGAGCTGTT

IL-1*β*	S	CCCTTGACTTGGGCTGT
	AS	CGAGATGCTGCTGTGAGA

TNF-*α*	S	CAGCCAGGAGGGAGAAC
	AS	GTATGAGAGGGACGGAACC

GAPDH	S	GTTGTGGCTCTGACATGCT
	AS	CCCAGGATGCCCTTTAGT

## Data Availability

The datasets used and analyzed during the current study are available from the corresponding author on reasonable request.
